# Book Review

**DOI:** 10.1120/jacmp.v4i4.2507

**Published:** 2003-09-01

## Abstract

**The Physics of Radiation Therapy** 3rd ed., by Faiz M. Khan, Lippincott, Williams & Wilkins, 2003. Price: $135.00. ISBN: 0‐7817‐3065‐1.

PACS number(s): 87.53.–j, 87.50.Gi, 87.52.Df

As the reviewer I debated between reviewing the book on a stand alone basis or in comparison to the very popular second edition of the text. As the book has become the standard text in the field over the last 19 years, I decided to write this Review as a comparison of the third edition to the second edition to see what changes have been made.

In general, the first thing one will note is that the third edition is 1 in. taller, and 1.5 in. wider. The quality of paper and printing appears to be the same as the second edition. Regrettably, this results in some of the radiographs which had been added to this edition not being clearly reproduced. The reader will find figures that sometimes appear before they are referenced and sometimes after they are referenced. An annoyance was to find all of the color figures for Chapters 19–23, which had been added as part of the new material, sandwiched in the center of the book in Chapter 14, presumably the result of some immutable aspect of the book publishing process.

The first seven chapters are almost identical with those of the second edition. Minor clarifications, editions, and corrections were made but one has to look for them. This results in some of the equipment photos being a bit dated, but the principles are still solidly presented.

Chapter 8 has been expanded to include such topics AAPM TG‐51 protocol for photon and electron calibration, the IAEA TRS 398 protocol, diode detectors, and GAF film dosimetry. These were nice additions and there are references to the original work in the topics.

Chapters 9 and 10 were essentially unchanged. In Chapter 11 the section on wedge design is now a bit dated, as no mention is made of the universal wedge which is used by one vendor or the dynamic wedge which is used by all vendors. A nice figure was added to help with the definition of GTV, PTV, CTV, TV, and IV Additional references were added to this chapter as well which are helpful.

Chapters 12 and 13 are essentially the same. A figure was added in Chapter 13 to clearly illustrate the penumbra differences between MLC and Cerobin block shaped fields.

Chapter 14, which includes output calibration, has been revised and updated. A section on planning algorithms is also added with the emphasis on the pencil beam algorithm. There are no changes to speak of in Chapters 15, 16, or 17.

Chapter 18, Total Body Irradiation, begins the really new material. This chapter is nicely done with the different geometries used (sitting, standing, supine on floor) discussed. The use of lung and other organ blocks is also described.

The steps of three dimensional (3D) conformal radiotherapy are outlined in Chapter 19, with specific reference made to ICRU 50, “Prescribing, recording, and reporting photon beam therapy.” The author gives a general scoping of the considerations involved and references to the original papers on the specific topics.

Chapter 20 is a good survey of intensity modulated radiation therapy (IMRT). Dr. Khan discusses the different leaf motion concepts, the types of machines which have been used, the commissioning tests involved with this new modality, patient treatment verification quality assurance, and the dose calculational algorithms used by some of the commercial systems.

Stereotactic Radiosurgery is discussed in Chapter 21 and equal treatment is given to the linac and radioisotope equipment. The equipment used to illustrate the section is still in use, but some is a bit dated.

In Chapter 22 the use of High Dose Rate irradiators is presented, and the procedural detail in this chapter is extensive. Discussed in the chapter are the uses for the modality, shielding design for dedicated HDR rooms, licensing requirements (USA) and the development of a quality management program. The emphasis is on compliance with the USA‐NRC guidelines, but the steps and procedures outlined would form a good basis of a program anywhere in the world.

Prostate implants are considered in Chapter 23. The author discusses the use of HDR, LDR, and permanent seed implants. The information on the seed parameters is limited, but references are given for information on others in use.

The new material added to the book concludes with a chapter (24) on Intravascular Brachy‐therapy. The author discusses the use of gamma and beta sources, radioactive stints, and liquid filled balloons. The dosimetry of each technique and their strengths and weaknesses are presented in a very even format.

In general, the new chapters that have been added are descriptive in nature with a few fundamental equations used to enhance specific sections. References are given to the original works which are more rigorous in their physics treatment of the topics. For those using the book as a text for course work, there are still no problems at the end of each chapter for the students to use to test themselves.

*James B. Smathers, PhD*

*ProfessorEmeritus, UCLA*

